
JOURNAL INFORMATION
==============================
NLM Title Abbreviation: Atlanta Med Surg J (1884)
NLM Journal ID: 101251623

Atlanta Medical and Surgical Journal (1884)

Publisher: Biomedical Journal Digitization Project

ARTICLE INFORMATION
==============================
PMCID: PMC9199293
PMID: 35829591
Article version: 1
Subjects: Editorial

The Bubonic Plague in Vienna

Print publication date: 1898 Dec
Volume: 15
Volume Series: New Ser
Issue: 10
First page: 685
Last page: 687
License: This work is free of known copyright restrictions. For more information, please see PMC Back Issue Digitization.
License URL: https://creativecommons.org/publicdomain/mark/1.0/

==============================

to usurp the special power which Christ had.' We feel it, further, to be our duty to point out that apart from evils which may result to individual members of the community from it, the Christian Science treatment is neither more nor less than a *fin-de-siècle* fad, a drawing-room cult, and that it is the counterpart of the culpable negligence which has brought the peculiar people of the less educated classes within the range and operation of the criminal law of the country."

---

### THE BUBONIC PLAGUE IN VIENNA.

THIS dreaded disease has claimed six victims in the Austrian capital as the result of experimental researches in the laboratory of Prof. Hermann Nothnagle. This, indeed, is an unfortunate occurrence, especially as it seems to have occurred as the result of scientific experiments. The *Philadelphia Medical Journal* has this to say:

"We have to record the melancholy intelligence of an unfortunate outbreak of bubonic plague in the Austrian capital. Some days ago, Herr Barisch, an employee in Professor Hermann Nothnagle's laboratory, while participating in investigations concerning the plague-bacillus, contracted the disease, and after a few days' illness succumbed on October 8th. The excitement occasioned by the announcement of this death was only augmented by the additional intelligence that the two nurses who had attended Herr Barisch themselves showed manifestations of the disorder. Almost immediately it was announced that Dr. Franz Herman Müller, who had been assiduous in his attentions to Herr Barisch, was attacked and became very ill, and that the wife of Herr Barisch and another assistant showed symptoms of the disorder. Popular excitement ran high, and the city is said to have been thrown on the verge of a panic by the death of Dr. Müller on October 23d.

As we go to press there have been reported six cases with two deaths. The apprehension of the inhabitants of the city seems not to have been allayed by the extraordinary precautions taken to prevent the spread of the disease. The body of Herr Barisch was securely wrapped in clothes saturated with germicides, placed in a double coffin, and hermetically sealed. The other patients were placed in an isolation-building and are attended by Dr. Poech, a volunteer physician and two Sisters of Charity. Neither egress nor ingress is permitted. Four other suspects have also been carefully isolated. All the animals used for experimental purposes in Professor Nothnagle's laboratory were killed and cremated. The government appointed a committee consisting of representatives of national and municipal bodies to devise ways and means to prevent the spread of the disorder. And finally, lest they should be taken unawares, a temporary hospital, consisting of several detached sheds, was hastily erected during the night, behind the Infectious-diseases Hospital.

It seems that constant association with highly virulent bacteria does beget in some a disregard of the attendant dangers, and doubtless after an investigation of the facts it will be found that the outbreak is clearly attributable to some neglected precaution. This is assuredly not as it should be, for the introduction of the Asiatic scourge into Europe, whether through the ordinary avenues of commerce or as the result of scientific investigation, is a most serious matter, and in either instance is to be equally deprecated. True, the disease is as yet limited to a half-dozen cases and will in all probability be eradicated, but its mere presence is food for serious thought. A most painful feature of the situation at present is the unscrupulous and uncompromising attitude of the antisemitic newspapers who are accusing the Jewish physicians of having introduced the plague into the city. It is feared that this appeal to the worst passions of the mob-populace may result in plague riots against the Jews in case the disease spreads; at the least, it may lead to a serious setback to the scientific investigation of the disorder. Dr. Müller was considered quite an authority on the plague. As a member of a committee consisting of himself, Dr. Albrecht, Dr. Ghon, and Dr. Poech, appointed by the Imperial-Royal Academy of Science, he proceeded in 1897 to Bombay and other points in India for the purpose of studying the plague. The committee returned some months ago to Vienna, and the report of its members has been noted in these columns."

---
